# Costs of resistance and infection by a generalist pathogen

**DOI:** 10.1002/ece3.1889

**Published:** 2016-02-17

**Authors:** Tad Dallas, Mathieu Holtackers, John M. Drake

**Affiliations:** ^1^ Odum School of EcologyUniversity of Georgia140 E. Green StreetAthensGeorgia30602; ^2^ Lambert High SchoolSuwaneeGeorgia30024

**Keywords:** *Daphnia*, inducible defenses, *Metschnikowia*, multihost pathogen, resistance costs

## Abstract

Pathogen infection is typically costly to hosts, resulting in reduced fitness. However, pathogen exposure may also come at a cost even if the host does not become infected. These fitness reductions, referred to as “resistance costs”, are inducible physiological costs expressed as a result of a trade‐off between resistance to a pathogen and aspects of host fitness (e.g., reproduction). Here, we examine resistance and infection costs of a generalist fungal pathogen (*Metschnikowia bicuspidata*) capable of infecting a number of host species. Costs were quantified as reductions in host lifespan, total reproduction, and mean clutch size as a function of pathogen exposure (resistance cost) or infection (infection cost). We provide empirical support for infection costs and modest support for resistance costs for five *Daphnia* host species. Specifically, only one host species examined incurred a significant cost of resistance. This species was the least susceptible to infection, suggesting the possibility that host susceptibility to infection is associated with the detectability and size of resistance cost. Host age at the time of pathogen exposure did not influence the magnitude of resistance or infection cost. Lastly, resistant hosts had fitness values intermediate between unexposed control hosts and infected hosts. Although not statistically significant, this could suggest that pathogen exposure does come at some marginal cost. Taken together, our findings suggest that infection is costly, resistance costs may simply be difficult to detect, and the magnitude of resistance cost may vary among host species as a result of host life history or susceptibility.

## Introduction

Pathogens are an important structuring force to host populations (Anderson and May 1978) and communities (Wood et al. 2007), with the potential to drive directional selection toward particular host genotypes (Duffy et al. 2012). Because pathogens have deleterious effects on host fitness, it is unsurprising that hosts respond to exposure through behavioral, immunological, and physiological pathways to reduce the negative effects of parasitism (Brace et al. 2015). Typically, these host responses result in reductions to host fitness through differential resource allocation. For instance, increased immune function in response to pathogen exposure can result in lower fecundity (Minchella 1985). Reductions to host fitness as a function of pathogen challenge can occur whether the host becomes infected (i.e., infection cost), or successfully evades infection (i.e., resistance cost). These costs are quantified as reductions in host fitness measures relative to unexposed, control hosts. Common host fitness measures used include host fecundity, body size, or survival (Zuk and Stoehr 2002; Hasu et al. 2009). The magnitude of these costs may depend on host genotype (Routtu and Ebert 2015), size of pathogen challenge, and environmental context, as seen in the dependence of the magnitude of resistance cost on the size of the pathogen challenge in a zooplankton (*Daphnia magna*) parasitized by a bacterial pathogen (*Pasteuria ramosa*; (Little and Killick 2007; Labbé et al. 2010)).

Despite the importance of these costs to host population structure and the spread of infectious disease, there is currently no consensus on the generality of resistance costs (Labbé et al. 2010). This is potentially a result of the diversity of host–pathogen interactions, or the range of deleterious effects pathogens may have on hosts (Møller et al. 1990). The lack of consensus is perhaps most pronounced in invertebrate hosts (Kraaijeveld et al. 2002; Ebert 2005a; Labbé et al. 2010), where linking pathogen exposure to immune function is difficult. While the existence of resistance costs in invertebrate host–pathogen interactions is unclear, evidence of infection costs is plentiful (Read 1994). For the purposes of this study, we define resistance costs as the negative effects resulting from pathogen challenge, but not infection, measured as differences in host fitness measures between hosts exposed to pathogen that do not become infected (hereafter referred to as “exposed‐uninfected”, or “resistant”) and unexposed, susceptible hosts (hereafter referred to as “control”). This most closely corresponds to what are considered activation costs of resistance (Auld 2013). Infection costs were defined as the reductions in host fitness as a result of pathogen infection, measured by comparing control hosts to infected hosts with respect to host fitness traits. Infection likely elicits a stronger reduction in host fitness by compounding the effects of pathogen exposure and infection. Presently, few studies have examined both resistance and infection costs simultaneously (but see (Ciota et al. 2011) for example). However, comparing the reductions in fitness between exposed‐uninfected (resistant) hosts and infected hosts could lead to an understanding of when resistance may be advantageous. Specifically, if the costs to host fitness are equal or greater in resistant hosts relative to infected hosts, resistance is unlikely to confer an advantage. However, if resistance is not very costly, as has been previously suggested (Labbé et al. 2010), then resistant individuals should have greater fitness than infected individuals.

Here, we addressed the impact of pathogen exposure and infection on host fitness using a generalist microparasite of *Daphnia* species. Many studies of resistance costs have focused on single host–pathogen pairs, which ignores the fact that pathogens tend to be able to infect multiple host species (Woolhouse et al. 2001), and hampers our ability to identify the potential host traits associated with the presence and size of resistance costs. We examined five zooplankton host (*Daphnia*) species for the presence of resistance and infection costs to a virulent fungal pathogen (*Metschnikowia bicuspidata*). Resistance and infection costs were measured in terms of three host fitness measures: total reproductive output, mean clutch size, and lifespan. We found a statistically significant resistance cost (i.e., fitness difference between exposed‐uninfected and control individuals) in only one host species, *D. pulicaria*, which is the least susceptible host species. Second, we found nearly universal costs of infection. However, there were no significant differences between exposed‐uninfected and infected host individuals. Taken together, we found limited support for significant costs of resistance, but qualitative evidence that exposed‐uninfected hosts had fitness values intermediate between infected and control hosts, suggesting that pathogen exposure can reduce host fitness, although the effects may be marginal. These nuanced costs of resistance, while not statistically significant when comparing control to exposed‐uninfected hosts, add an interesting dimension, and a potential avenue for quantifying resistance costs. Specifically, the relative difference between exposed‐uninfected hosts and both control and infected hosts contains information on the cost of resisting or tolerating a pathogen infection.

## Methods

### Origin and maintenance of hosts and pathogen

Monoclonal lines of five *Daphnia* species (*D. ambigua*,* D. dentifera*,* D. laevis*,* D. mendotae*, and *D. pulicaria*) were maintained in experimental media best suited for host survival (different proportions of EPA hardwater media (US Environmental Protection Agency, 2002) and deionized water, *D. ambigua*, 20%; *D. laevis* and *D. mendotae*, 33%; *D. pulicaria*, 50%), except for *D. dentifera*, which were maintained in dilute pondwater (50%). Host species clones were laboratory‐reared for many generations before this experiment, but were originally cultured from a small pond in Victoria Bryant State Park (*D. ambigua*), a Michigan Lake (provided by Meghan Duffy of University of Michigan; *D. dentifera*), Ellenton Bay (Aiken, SC; *D. laevis*), a small pond in Northern IL (*D. mendotae*), and Oneida Lake (clone #29, provided by Hairston Lab at Cornell; *D. pulicaria*). All host cultures were fed 50 *μ*L of a 2 g/L suspension (equivalent to 1 mg/L algal dry weight) of pulverized blue‐green algae (*Spirulina* sp.), and kept on the laboratory benchtop under constant overhead lighting. Previous exposure of host clones to *M. bicuspidata* could potentially alter the expression of resistance or infection costs, but the data on previous pathogen exposure were not available for the clones studied here. However, laboratory clones were raised under laboratory conditions for more than 20 generations before their first pathogen exposure, which reduces the possibility of potential legacy effects of pathogen exposure.

The fungal pathogen used in this study (*M. bicuspidata*) was originally isolated from *D. dentifera* in Michigan lakes (provided by Meghan Duffy). The pathogen was cultured *in vivo* by exposing *D. dentifera* to infectious spores and harvesting the spores by homogenizing infected animals in deionized water. Parasite fitness may be altered by host genotype, but no heritable variation exists in the fungal pathogen studied (Searle et al. 2015). This means that rapid pathogen evolution in response to hosts is unlikely, but also that the host genotype used to culture the pathogen could influence pathogen infectivity. To account for this, the pathogen was always cultured in a single clone of *D. dentifera*, and hosts were only exposed to the pathogen a single time (i.e., uninfected hosts from one round of pathogen exposure were not used subsequently). The host range of the fungus is unknown, but includes a variety of both terrestrial and marine organisms (Codreanu and Codreanu‐Balcescu 1981; Moore and Strom 2003). The pathogen is environmentally transmitted during host host feeding (Ebert 2005b; Hall et al. 2009). Pathogen spores pierce the gut wall, and proliferate inside the host until host death causes the release of pathogen spores into the environment. Infection is easily diagnosed, as spores form opaque clusters in the transparent host (see (Penczykowski et al. 2014) or journal cover image from (Duffy et al. 2012)).

### Experimental design

To remove the confounding effects of host age and maternal environment, we sequentially isolated offspring from parthenogenetic females raised in isolation to obtain individuals of known age and maternal environment. Keeping maternal environmental conditions fairly uniform, and randomly placing individuals in experimental groups serves to reduce any effect of maternal environment. Sequential isolation was performed for three generations before hosts were used in experiments, and the resulting offspring of this process were randomly assigned to exposure treatment. Host age may influence within‐host pathogen competition (Izhar et al. 2015), as host immunity may change as a function of age, and fitness costs since fitness and energy allotment to growth or reproduction vary over the lifespan of the host (Izhar and Ben‐Ami 2015). To account for this, we sequentially isolated *Daphnia* hosts as described above for 12 days, isolating six uninfected individuals per species per day (*n *=* *72 hosts of known age per species examined), creating a uniform age gradient for all host species. Animals were placed individually in 50 mL of appropriate media, and either exposed to pathogen (200 pathogen spores/mL) or a slurry of crushed *D. dentifera* as a control (sham) inoculum. This was performed because the pathogen inoculum was created by crushing infected hosts, and the presence of crushed *Daphnia* may signal an alarm response from conspecifics.

Experimental individuals were monitored daily for offspring production, mortality, and ephippia (resting egg) production. Infections are typically unobservable before 7 days postinfection challenge, and mortality typically occurs after 12 days or more. Infection was assessed visually daily from day seven onward and confirmed at death by examining *Daphnia* hosts using a compound microscope (400× magnification). In this approach, *Daphnia* hosts were crushed between glass microscope slide and coverslip and examined thoroughly for the presence of pathogen spores, which normally average over 10,000 per infected host (Dallas and Drake 2014). In our experiment, none of the control hosts became infected, and not all hosts exposed to pathogen spores became infected. One host species, *D. dentifera*, was excluded from the analyses as a result of excessively high host mortality. However, we reproduce manuscript plots with the inclusion of *D. dentifera* in the Supplementary Materials.

We quantified costs as relative changes in three host fitness measures; total reproduction, mean clutch size, and lifespan. Total reproductive output (total number of offspring produced per host individual) and mean clutch size (mean number of offspring per reproduction event) were both measured after the host had been exposed to the pathogen (or control inoculum). Host lifespan was measured as the total number of days from host birth to host death.

### Statistical analysis

To assess differences among host exposure classes (i.e., control, exposed‐uninfected, and infected), we used Kruskal–Wallis rank tests. Nemenyi *post hoc* tests were used to examine pairwise differences between exposure classes. This analysis allowed for the separate determination of costs of resistance (control compared with exposed‐uninfected host fitness), and costs of infection (control compared with infected host fitness). Further, this approach also enabled us to compare the rank distributions of exposed‐uninfected hosts to infected hosts, thereby providing insight into how costly resistance is compared to infection. All analyses were performed in R (R Core Team, 2015), and Nemenyi *post hoc* tests were performed using the PMCMR package (Pohlert 2015).

## Results

### Costs of pathogen infection

Host fitness, measured as total reproduction, mean clutch size, and lifespan, was systematically reduced as a function of pathogen infection (Table 1), suggesting that microparasite infections were costly. Specifically, infection costs, measured as fitness differences between control (unexposed) and infected host individuals, were nearly universally significant (Table 2), resulting in sizable reductions to host reproductive output ($\bar{\mu_{c}-\mu_{i}}$ = 18.6 neonates), mean clutch size ($\bar{\mu_{c}-\mu_{i}}$ = 1.1 fewer neonates per clutch), and lifespan ($\bar{\mu_{c}-\mu_{i}}$ = 6.8 days). The consistent finding of infection costs was not found for *D. dentifera*, which was excluded from the analyses as a result of enhanced mortality early in the experiment (see Supplementary Material).

**Table 1:** Mean and standard error for fitness measures (reproductive output, lifespan, and mean clutch size) for control, exposed‐uninfected, and infected individuals

Host	Infection status	*n*	Reproduction	Lifespan	Mean clutch size
*D. mendotae*	Control	36	14.89 (2.57)	24.58 (1.53)	2.82 (0.36)
	Exposed‐uninfected	2	10.50 (0.50)	19.50 (0.50)	3.50 (0.17)
	Infected	34	3.47 (0.77)	16.68 (0.74)	1.60 (0.24)
*D. ambigua*	Control	36	31.06 (4.08)	24.67 (1.66)	3.94 (0.32)
	Exposed‐uninfected	10	16.80 (4.01)	18.90 (1.69)	3.39 (0.62)
	Infected	26	9.77 (1.48)	17.96 (1.06)	2.65 (0.30)
*D. laevis*	Control	36	36.69 (3.85)	25.53 (1.57)	4.52 (0.35)
	Exposed‐uninfected	12	16.58 (4.22)	18.17 (1.22)	3.49 (0.49)
	Infected	24	12.33 (2.67)	19.83 (1.28)	3.05 (0.35)
*D. pulicaria*	Control	36	35.92 (3.64)	32.83 (1.99)	4.29 (0.30)
	Exposed‐uninfected	36	14.56 (1.88)	22.11 (1.14)	3.33 (0.35)
	Infected	0	–	–	–

Host species are ordered from most to least susceptible to infection by *M. bicuspidata*.

**Table 2:** The costs of resistance and infection to a generalist microparasite

Host	Covariate	*μ*_c–_ *μ*_r_	*K*_*cr*_	*P*_*cr*_	*μ*_c–_ *μ*_i_	*K*_*ci*_	*P*_*ci*_
*D. mendotae*	Reproduction	4.39	0.77	0.848	11.42	5.03	**0.001**
	Lifespan	5.08	0.99	0.764	7.91	5.89	*<* **0.0001**
	Mean clutch size	−0.68	1.45	0.560	1.22	3.49	0.036
*D. ambigua*	Reproduction	14.26	1.77	0.423	21.29	5.06	**0.001**
	Lifespan	5.77	2.72	0.133	6.71	4.42	**0.005**
	Mean clutch size	0.55	1.25	0.648	1.29	4.23	**0.008**
*D. laevis*	Reproduction	20.11	3.50	0.036	24.36	5.58	*<* **0.0001**
	Lifespan	7.36	4.14	**0.010**	5.69	4.33	**0.006**
	Mean clutch size	1.02	2.65	0.146	1.46	4.50	**0.004**
*D. pulicaria*	Reproduction	21.36	6.00	*<* **0.0001**	–	–	–
	Lifespan	10.72	6.43	*<* **0.0001**	–	–	–
	Mean clutch size	0.91	3.46	**0.014**	–	–	–

Costs are measured as reductions in lifetime reproduction, mean clutch size, and lifespan. Differences between unexposed control (*c*) hosts and both infected (*i*) and resistant (exposed‐uninfected; *r*) hosts. Mean group differences are provided in columns *μ*_*c–*_ *μ*_*i*_, where *i* corresponds to either resistant (*r*) or infected (*i*) hosts. Significance (*P*‐values are in bold) was assessed at *α* = 0.0167 to correct for multiple comparisons among pathogen exposure classes (i.e. control, exposed‐uninfected, and infected).

### Costs of resistance to pathogen

**Figure 1:**
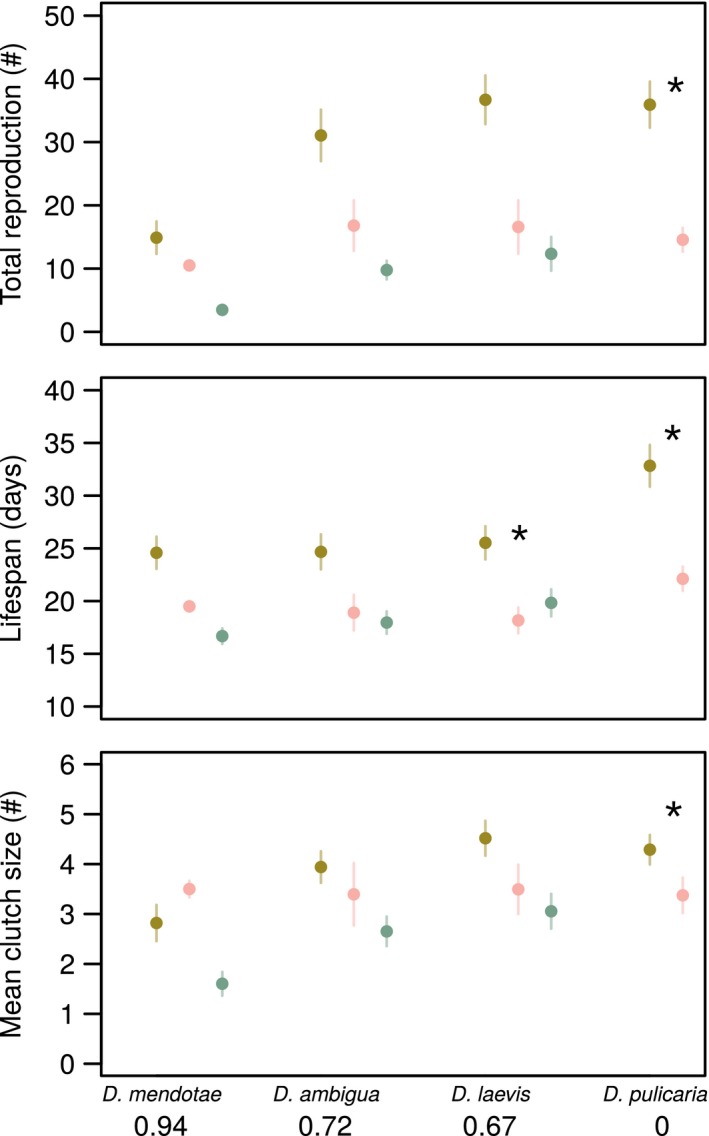
Significant costs of resistance (denoted with an asterisk; *), and infection (universal except for mean clutch size of *D. mendotae*) with respect to three host fitness measures (mean ± 1SE). Mean clutch size and total reproduction were quantified as the number of offspring per clutch and the total number of offspring an individual produced after infection challenge. Lifespan was scored as total lifespan of the host. Host susceptibility, defined as the fraction of hosts exposed to the pathogen that became infected, is given in parentheses next to the host species name.

Meanwhile, exposure to pathogen without infection did not cause a significant reduction in host fitness for a majority of the host species and fitness measure combinations (Table 2), suggesting that resistance in the *Daphnia*–microparasite system is not costly. However, significant resistance costs were observed for *D. laevis* with respect to lifespan, and in all fitness measures for *D. pulicaria* (Table 2). This host species does not become infected by the pathogen. For the other three species examined, exposed‐uninfected individuals did not differ in fitness relative to control hosts or infected hosts, suggesting that exposed‐uninfected hosts have fitness values intermediate to hosts not exposed to the pathogen, and hosts that become infected (Fig. 1). While not statistically significant, pathogen exposure reduced mean host fitness, in terms of average host reproductive output ($\bar{\mu_{c}-\mu_{r}}$ = 13.5 fewer total neonates), clutch size ($\bar{\mu_{c}-\mu_{r}}$ = 0.08 fewer neonates per clutch), and lifespan ($\bar{\mu_{c}-\mu_{r}}$ = 6.9 days).

### Does host age influence costs?

Host age was strongly and positively related to host fitness measures, as older hosts at the time of pathogen exposure produced more offspring, had larger mean clutch sizes, and had longer lifespans relative to hosts that were younger at the time of pathogen exposure. However, we found little evidence for variation in resistance or infection costs as a function of host age at the time of pathogen exposure, although this relationship was significantly positive in *D. laevis* hosts when costs were measured in terms of lifespan or mean clutch size (see Supplementary Materials).

## Discussion

Responding to a pathogen challenge is expected to reduce host fitness by diverting limited host resources toward pathogen resistance (i.e., an inducible cost). However, consistent evidence for resistance costs remains sparse, both in laboratory (Ebert et al. 1998; Labbé et al. 2010; Garbutt et al. 2014) and field (Auld et al. 2013) studies. Here, we attempted to identify resistance and infection costs for a generalist pathogen capable of infecting numerous *Daphnia* host species. We provide evidence that fungal pathogen infections come at a fitness cost to all susceptible host species, but that the fitness consequences of pathogen exposure were more nuanced. Specifically, significant resistance costs were only observed in *D. pulicaria*, a completely resistant host species. However, exposed‐uninfected (resistant) hosts had fitness values intermediate between control hosts and infected hosts. This suggests that pathogen resistance still comes at a price, although this difference is insignificant based on our limited sample size. Neither resistance nor infection costs varied as a function of host age at the time of pathogen exposure, although previous studies have found an age‐dependent cost in *Daphnia* parasitized by a castrating bacterial pathogen (Izhar and Ben‐Ami 2015). Taken together, these results support previous findings (Labbé et al. 2010) suggesting that resistance does not come at a high cost in *Daphnia*–microparasite interactions, provide one of the first examinations of costs associated with a multihost pathogen, and suggest that host susceptibility may be related to the size of resistance costs.

Perhaps coincidentally, species incurring the largest costs of resistance were also the least likely to become infected by the pathogen. Different clonal lines of *D. pulicaria* have also demonstrated this resistance (*unpublished data*, and Dallas et al. in press). Our ability to make broad generalizations about the relationship between host species susceptibility and resistance costs is limited by the examination of single representative clones of each *Daphnia* species. However, we found a consistent decline in magnitude of resistance cost with increasing host species susceptibility to infection (see Supplementary Materials), which was significant when costs were measured in terms of change in host lifespan between control and exposed‐uninfected hosts. Potentially the most obvious explanation for this relationship is that less susceptible species are less susceptible because they are able to mount an effective, though costly, behavioral or immunological response. Behaviorally, hosts could reduce feeding, which would reduce pathogen transmission, but would also reduce fitness through resource limitation. This behavioral response could also explain previous findings in natural systems, in which populations of *D. dentifera* exhibited a negative relationship between pathogen transmission rate and host birth rate (Auld et al. 2012). This observed cost of resistance could be a result of the close relationship between *Daphnia* species feeding rate and both pathogen transmission and host birth rate. Understanding both the behavioral and immunological mechanisms contributing to resistance costs in multihost pathogens is an important, but as yet unexplored, topic.

Host age has been hypothesized to influence the size of the host response to pathogen exposure. This has been shown previously in a castrating bacterial pathogen of *Daphnia* (Izhar and Ben‐Ami 2015), as younger hosts had higher transmission, shorter time until castration, and higher pathogen fitness (i.e., infection intensity). There are at least two separate reasons for the difference in detectability of age‐depdendent costs. First, bacterial pathogens, especially castrating bacterial pathogens, could elicit a different response than fungal pathogens. This is because bacterial castrating pathogens, like the pathogen examined by (Izhar and Ben‐Ami 2015), have strong effects on host fitness, and often exhibit co‐evolutionary relationships with hosts (Ebert 2008). Therefore, the existence of age‐dependent costs could be a result of the type of pathogen examined, and the relative virulence of the pathogen. Second, the current study examined a narrow age range (1–12 days old) based on the survival of hosts in the laboratory. Izhar and Ben‐Ami (2015) examined a longer‐lived *Daphnia* host species, and three host ages (5, 15, and 30 days old at the time of pathogen exposure). The mean lifespan of hosts, regardless of pathogen exposure, was <30 days, likely a result of experimental conditions (e.g., feeding live algae versus a *Spirulina* suspension). A final explanation could be the effects of pathogen dose or environmental conditions (apart from resources as described above). This explanation could explain not just the lack of detected age dependence, but also potentially the lack of detectability of resistance costs in invertebrate systems.

There is currently no consensus about why resistance costs are detectable in some systems, and apparently absent in others, especially for invertebrate pathogens (Ebert et al. 1998; Labbé et al. 2010; Garbutt et al. 2014). Environmental stress (Sandland and Minchella 2003) and evolutionary history (Little et al. 2002) have both been invoked as factors potentially obscuring (or promoting) the detection of costs. There are many other potential causes for the failed detection of resistance costs in *Daphnia*, including the use of an immutable trait to quantify cost, and a limited understanding of invertebrate immunology (Little et al. 2005). The focus on single species host–pathogen systems also limits our understanding of resistance costs. We attempted to address this by examining multiple host species, allowing the potential for a more mechanistic examination of resistance costs. The relationship between aspects of host species (e.g., phylogenetic relatedness, susceptibility to infection, life history traits) and the magnitude of resistance costs could provide insights into why these costs are observed in some host—pathogen combinations and not in others. Lastly, because resistance costs may be mediated by changes to host phenotype, life history, behavior, or immunology (Rigby et al. 2002), it is possible that costs are incurred without being detected. This may explain, in part, the mixed support for resistance costs in many animal systems, including *Daphnia* (this study; (Labbé et al. 2010)), birds (Norris and Evans 2000), and amphibians (Cheatsazan et al. 2013).

Investigations of resistance and infection costs incorporating the effects of environment, differential pathogen exposure (i.e., number, duration, and dose of pathogen exposure), and host life history may provide a more detailed understanding of when a host response to pathogen exposure can be costly. By examining multiple host species, we provide little evidence for resistance costs in *Daphnia*—fungal pathogen interactions, but overwhelming support for costs of infection. Resistant individuals still had reduced fitness, representing an intermediate point between unexposed control hosts and infected hosts, suggesting that resistance may still come at a cost, but that this cost may be difficult to detect. Future studies of resistance costs to multihost pathogens in the presence of environmental stressors are necessary for the development and testing of hypotheses related to the expression and magnitude of resistance costs. Further, integrating resistance costs into epidemiological models and experiments may be critical to developing an understanding of pathogen‐mediated host competition, host community structure, and host–pathogen interactions in general.

## Conflict of Interest

None declared.

## Supporting information


**Table S1**. Mean and standard error for fitness measures (reproductive output, lifespan, and mean clutch size) for control, exposed‐uninfected, and infected individuals.
**Figure S1.** Distribution of lifespan by host species.
**Figure S2.** Costs of resistance and infection to a generalist fungal pathogen.
**Figure S3.** Resistance costs along a gradient of host age at pathogen exposure.
**Figure S4.** Infection costs along a gradient of host age at pathogen exposure.
**Figure S5.** Resistance costs scale with host susceptibility.
**Figure S6.** Resistance costs scale with host susceptibility, but not when *D. dentifera* is included.
