# Supplementary material for: Costs of resistance and infection by a generalist pathogen
Source: Ecol Evol. 2016 Feb 17;6(6):1737–44. doi: 10.1002/ece3.1889 (PMC4757773; doi:10.1002/ece3.1889)
Supplement: Supplementary file 1 — Table S1. Mean and standard error for fitness measures (reproductive output, lifespan, and mean clutch size) for control, exposed‐uninfected, and infected individuals. Figure S1. Distribution of lifespan by host species. Figure S2. Costs of resistance and infection to a generalist fungal pathogen. Figure S3. Resistance costs along a gradient of host age at pathogen exposure. Figure S4. Infection costs along a gradient of host age at pathogen exposure. Figure S5. Resistance costs scale with host susceptibility. Figure S6. Resistance costs scale with host susceptibility, but not when D. dentifera is included. [file ECE3-6-1737-s001.pdf]

Supplementary Material for: Costs of resistance and infection by a generalist pathogen

Tad Dallas[1,3], Mathieu Holtackers [2], and John M. Drake[1]

Affiliations:

1. University of Georgia, Odum School of Ecology, 140 E. Green Street, Athens GA, 30602
2. Lambert High School, Suwanee, GA, 30024
3. Corresponding author: `tdallas@uga.edu`

The aberrant fifth host species In the main text, we report on the results from four *Daphnia* host species, but the experiment actually included a fifth species (*D. dentifera*). However, this host species suffered high mortality, and had an average lifespan of around 15 days, and twenty individuals surviving for fewer than ten days (Figure S1). Apart from mortality, 44% of hosts did not reproduce, and clutch sizes of those that did successfully reproduce tended to be small. As a result of high mortality, replication was insufficient to test for resistance and infection costs. Because of all the reasons above, we excluded *D. dentifera* from analysis. However, for completeness, we recreate Table 1 (see Table S1) and Figure 1 (see Figure S2) from the main text here with *D. dentifera* included.

The effect of host age at exposure on the magnitude of resistance and infection costs Hypothetically, older hosts should respond to pathogen challenge differently than younger hosts, as resistance should tradeoff with host fitness, which is intrinsically related to host age for most organisms. Therefore, older hosts would be expected to not mount a large resistance response. To examine the effect of host age on resistance and infection costs, we fit linear models to the relationship between host

age at pathogen exposure and the relative difference in total reproduction, mean clutch size, and lifespan between exposed-uninfected and infected individuals of the same age (infection costs), and exposed-uninfected and resistant hosts of the same age (resistance costs). We found no evidence that host age at pathogen exposure influenced resistance (Figure S3) or infection (Figure S4) costs, except for a positive relationship between host age and the magnitude of resistance cost in terms of total reproduction for *D. pulicaria*. This means that there was a greater difference in total reproduction between resistant and control hosts when hosts were older. The fact that *Daphnia pulicaria*, a host that has never, to our knowledge, been observed to be infected, incurred such a great cost of resistance, is curious and seemingly maladaptive, when the probability of becoming infected is low (or null).

The potential relationship between host susceptibility and resistance costs

The relative per-species difference between exposed-uninfected host individuals and control individuals (i.e. resistance cost size) was dependent on host species susceptibility (Figure S5), but not when including data from *D. dentifera* (Figure S6). This difference was calculated by sampling control and resistant hosts of a single species, truncating the control host samples to be the same length as the resistant host samples, and taking the difference between the means. This was performed 1000 times for each host species and fitness metric combination, which allowed the plotting of both mean and standard deviation of the mean difference between control and resistant hosts. The use of a single clone of each host species examined makes interspecific comparisons difficult, as there could be large intraspecific variation in physiological responses to pathogen exposure. We therefore do not make any claims regarding the generality of the relationship between resistance cost and host susceptibility. However, this is an interesting open question, as the answer could potentially provide a more mechanistic or evolutionary perspective on interspecific differences in resistance costs. Specifically, perhaps host species are less susceptible because they mount such a large resistance effort. Understanding the mechanistic basis of interspecific variation in resistance costs is an interesting, and currently largely unexplored research area.

Tables

Table S1: Mean and standard error for fitness measures (reproductive output, lifespan, and mean clutch size) for control, exposed-uninfected, and infected individuals. Host species are ordered from most to least susceptible to infection by *M. bicuspidata*.

**Host**	**Infection status**	**n**	**Reproduction**	**Lifespan**	**Mean clutch size**
D. mendotae	control	36	14.89 (2.57)	24.58 (1.53)	2.81 (0.36)
	exposed-uninfected	2	10.5 (0.50)	19.50 (0.50)	3.50 (0.17)
	infected	34	3.47 (0.77)	16.68 (0.74)	1.61 (0.23)
D. ambigua	control	36	31.67 (4.08)	24.67 (1.66)	3.88 (0.31)
	exposed-uninfected	10	16.80 (4.01)	18.90 (1.69)	3.39 (0.62)
	infected	26	10.85 (1.55)	17.96 (1.06)	2.67 (0.26)
D. dentifera	control	36	7.97 (1.67)	15.53 (1.21)	2.22 (0.40)
	exposed-uninfected	11	10.00 (2.64)	16.27 (2.12)	3.10 (0.49)
	infected	25	5.08 (1.45)	14.60 (1.35)	1.75 (0.39)
D. laevis	control	36	36.97 (3.90)	25.53 (1.57)	4.48 (0.35)
	exposed-uninfected	12	23.83 (6.89)	19.50 (0.90)	4.87 (1.18)
	infected	24	12.83 (2.64)	19.83 (1.28)	3.02 (0.34)
D. pulicaria	control	36	35.92 (3.64)	32.83 (1.99)	4.29 (0.30)
	exposed-uninfected	36	14.56 (1.88)	22.11 (1.14)	3.33 (0.35)
	infected	0	–	–	–

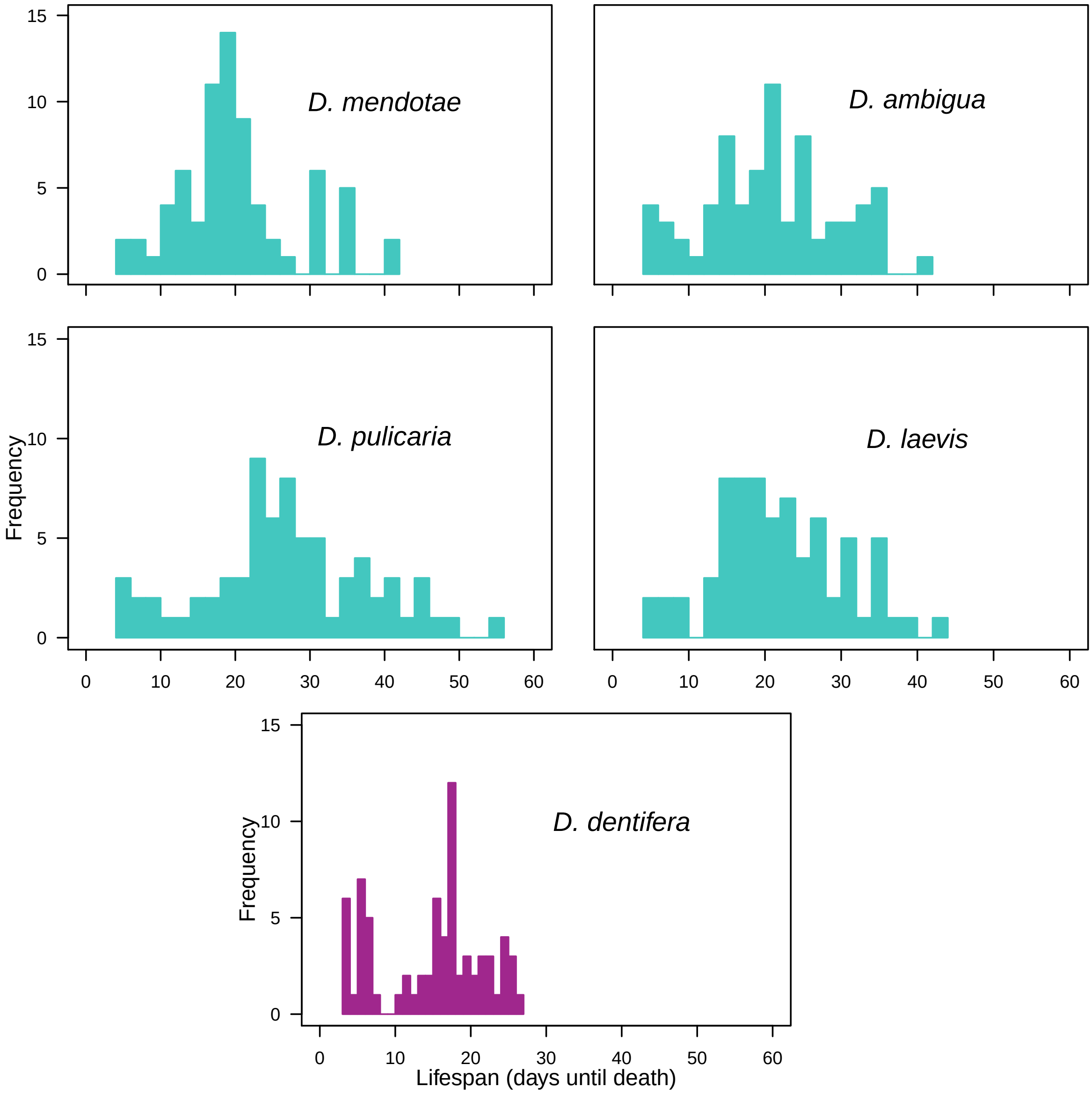

Figure S1: **Distribution of lifespan by host species**. High mortality in *D. dentifera* resulted in the exclusion of this species from the analysis of the main text. Note the clump of *D. dentifera* hosts (bars are colored fuschia to highlight panel) with lifespans less than 10 days. Further, no *D. dentifera* host lived longer than 30 days.

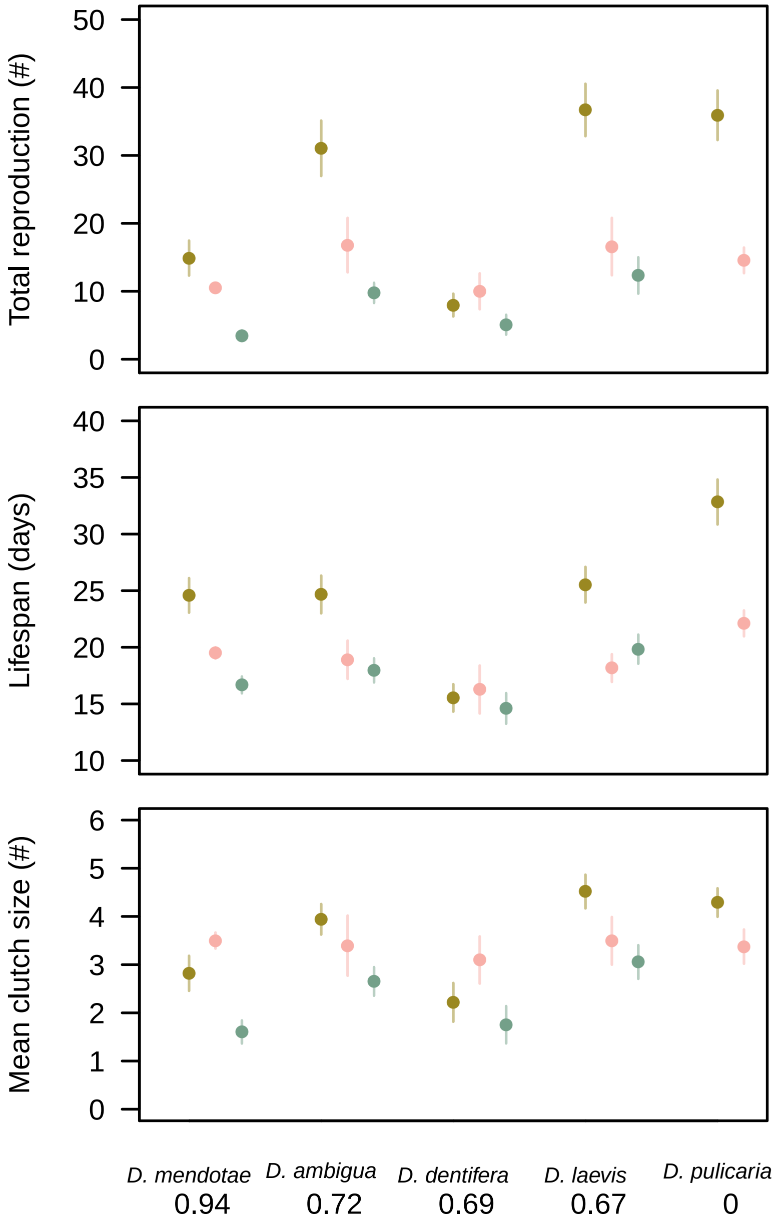

Figure S2: **Costs of resistance and infection to a generalist fungal pathogen**. This is the same as the main text Figure 1, but includes *D. dentifera*, who was excluded because of high mortality observed.

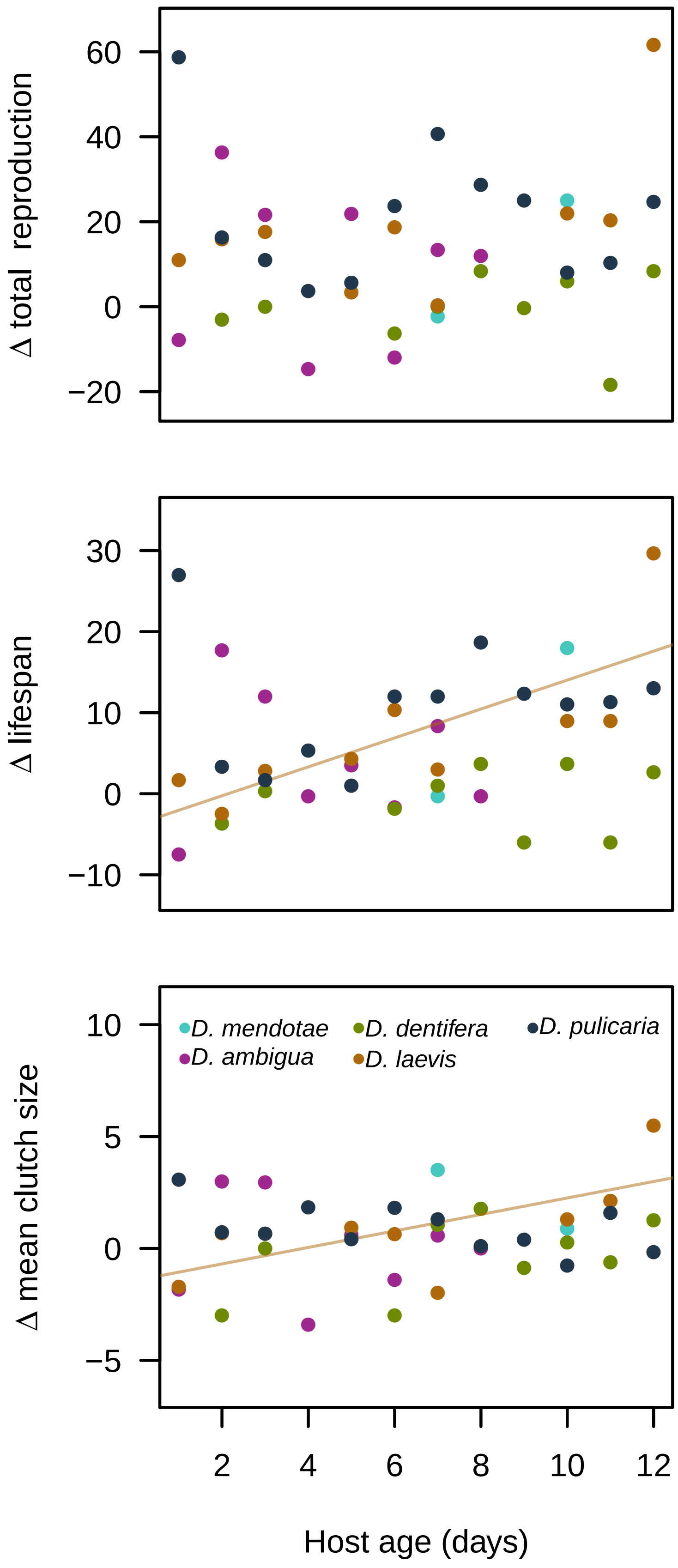

Figure S3: **Resistance costs along a gradient of host age at pathogen exposure**. Host age at pathogen exposure significantly influenced resistance costs in one host (*D. laevis*) for two (lifespan and mean clutch size) of the three fitness measures examined.

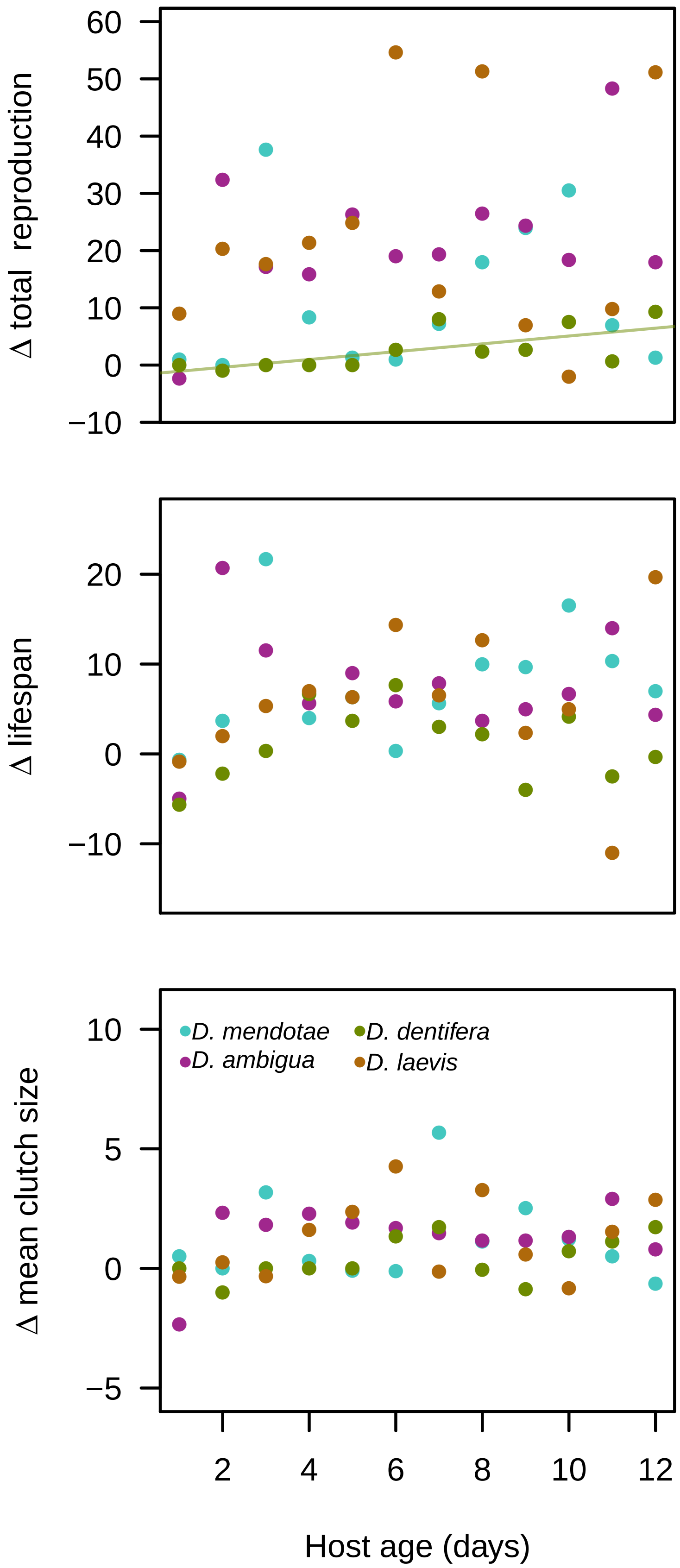

Figure S4: **Infection costs along a gradient of host age at pathogen exposure**. Infection costs were unrelated to host age at pathogen exposure, except for reproduction of *D. dentifera*, though this host was excluded from analyses, and the age effect on change in total reproduction is small.

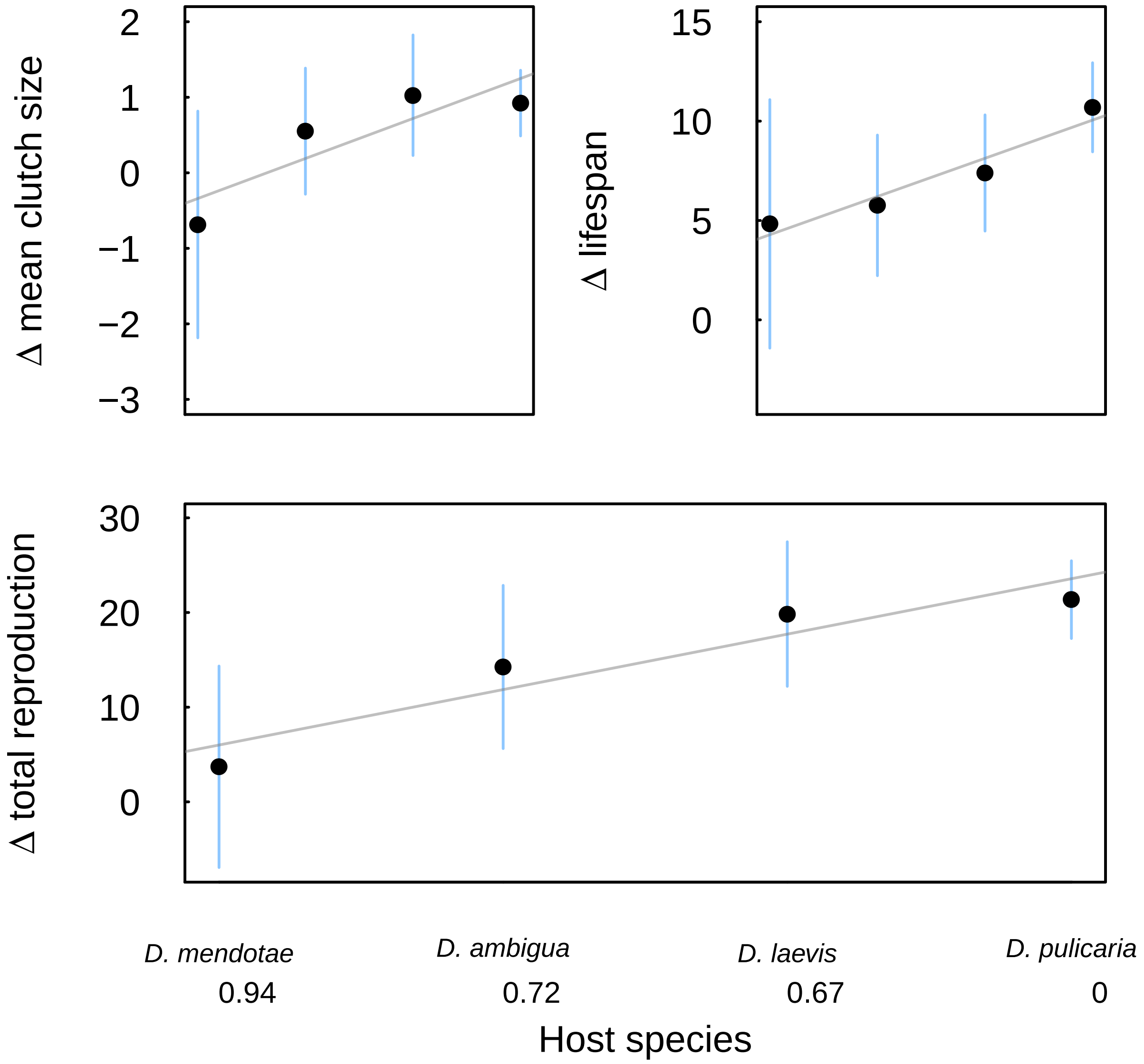

Figure S5: **Resistance costs scale with host susceptibility**. The difference between means (calculation described above) is plotted for 1000 bootstrapped samples. Plotted points are mean differences +- 1 standard deviation. Grey lines are linear models for illustrative purposes, though the relationship is significant for lifespan (adj. $R^2 = 0.911$, $p = 0.03$).

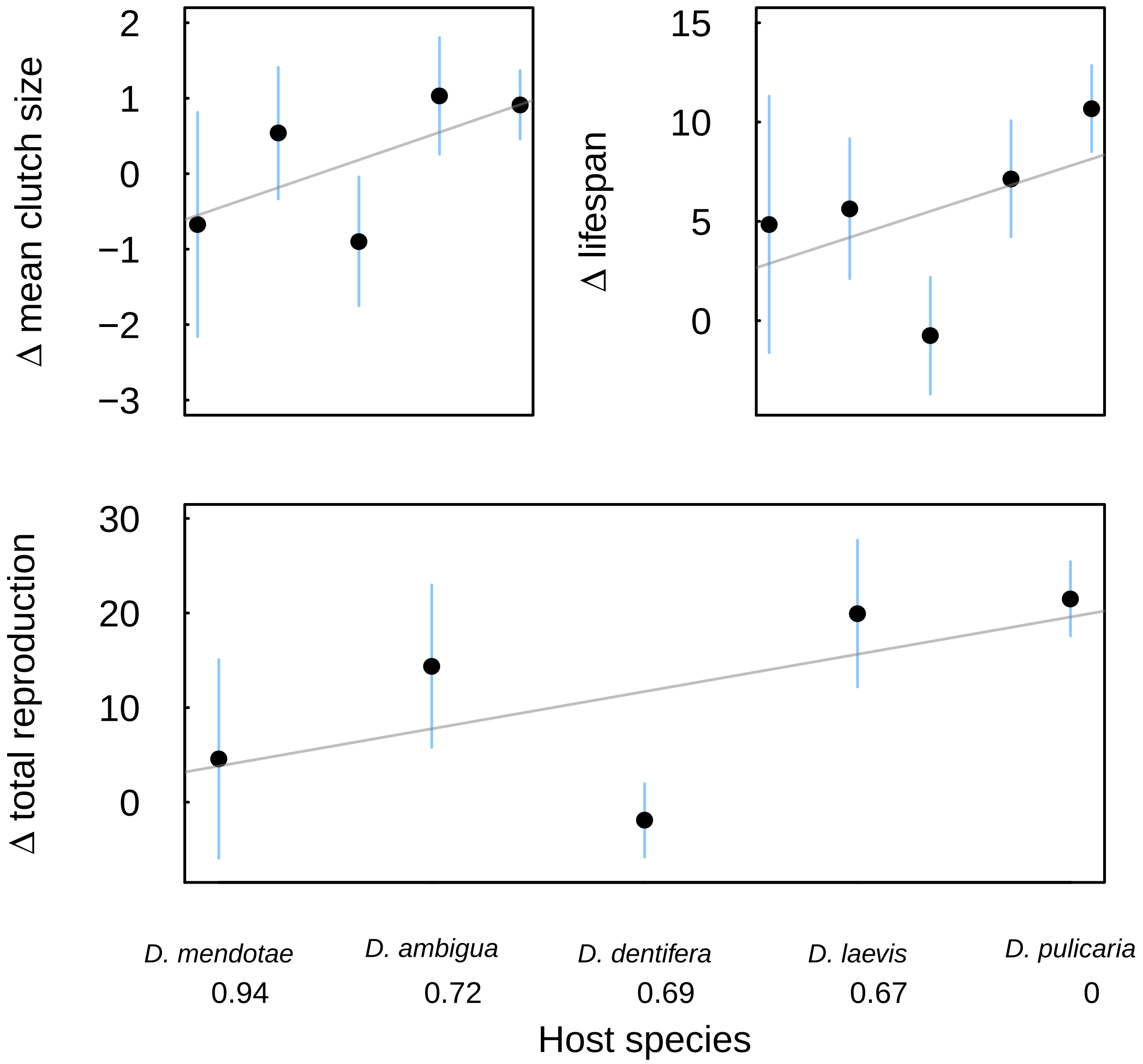

Figure S6: **Resistance costs scale with host susceptibility, but not when *D. dentifera* is included**. The difference between means (calculation described above) is plotted for 1000 bootstrapped samples. Plotted points are mean differences +- 1 standard deviation. Here, we include *D. dentifera*, though this host species suffered enhanced mortality early in the experiment, and was subsequently removed from our analysis.
